# Clinico-pathological profile of sinonasal masses: an experience in national ear care center Kaduna, Nigeria

**DOI:** 10.1186/1756-0500-3-186

**Published:** 2010-07-09

**Authors:** Aminu Bakari, Olushola A Afolabi, Adeyi A Adoga, Aliyu M Kodiya, Babagana M Ahmad

**Affiliations:** 1 National Ear Care Center, PMB 2438, No 3 Golf/Independence Way, Kaduna, Nigeria

## Abstract

**Background:**

The presence of a mass in the nose and paranasal sinuses may seem to be a simple problem; however it raises many questions about the differential diagnosis. The aim of this study is to evaluate the clinico-pathological profile of sinonasal masses in our environment

This is a retrospective analytical review of all the patients with sinonasal masses that presented to the national ear care center, Kaduna over a six year (2003-2008) period. Their biodata, clinical profile and histological diagnoses were analyzed.

**Findings:**

A total of 76 patients were analyzed, age range 5 to 64 yrs with a mean age of 33.3 yr median and modal age of 35.00 (SD = 13.1 ± 1.5). Majority of the patients were in the age groups 21-50 yrs. There were 34 male and 42 female with M: F ratio of 1:1.2. The main presenting symptoms are nasal blockage 97.4% and rhinorrhea 94.7%. It was bilateral in 34 (44.7%), left side in 24(31.6%) and right side in 18(23.7%) patients. The commonest clinical diagnoses were simple nasal polyp 47(61.8%) and antrochoanal polyp 10(13.2%). About 59 (77.6%) were benign, 2 (2.6%) were malignant and 15 (19.7%) were lost to follow up. The commonest histological diagnosis is simple inflammatory nasal polyp in 28 (36.8%) patients and the least was nasal capillary hemangioma 2 (2.6%). About 55(72.4%) patients had surgical treatment.

**Conclusions:**

Nasal obstruction and rhinorrhea are the commonest symptoms of presentation, simple inflammatory nasal polyp is still the commonest histological pattern seen in our environment, and surgery is still the best modality of treatment for benign tumor thus the need for advocacy for early recognition and referral to the ENT surgeon.

## Introduction

The presence of a mass in the nose and paranasal sinuses may seem to be a simple problem; however it raises many questions about the differential diagnosis. Nasal polyps (NPs) as part of sinonasal masses (SNM) have been a medically recognized condition since the time of the ancient Egyptians and their removal with a snare was described by Hippocrates, a method which persisted well into the second half of the 20th century [1].

Simple nasal polyps are round, smooth, soft, translucent, yellow or pale glistening structures attached to the nasal or sinus mucosa by a relatively narrow stalk or pedicle. They are non-tender and displaced backwards on probing. These features clinically distinguish them from the turbinates, which are sometimes assumed to be nasal polyps by the less experienced [2]. Classically they are caused by a combination of allergy and infection [3]. Turbinates will shrink on application of vasoconstrictors but polyps will not shrink [2].

Polyps are a common cause of nasal obstruction in the adult, while the diagnosis in children is so rare (0.1%) as to be questionable [1]. In the general population, the prevalence of NP is considered to be around 4% [3]. In cadaveric studies, this prevalence has been shown to be as high as 40% [4]. It predominantly affects adults usually those older than 20.

Benign neoplasia of the nose and paranasal sinuses is relatively not uncommon [4]. Cancers of the nose and paranasal sinuses account for less than 1% of all malignancies and about 3% of all head and neck cancers [5]. It has a geographic tendency to affect the African, the Japanese, and the Arab [5]. It is rarer in Western Europe and America [5].

The aim of this study is to evaluate the clinico-pathological profile of sinonasal masses (SNM) seen during the study period and to draw attention to the fact that not all cases of nasal obstruction/discharge are due to chronic infective/allergic sinusitis.

## Method

This is a retrospective analytical review of all the patients with sinonasal masses that presented to the national ear care center, Kaduna over a six year period (2003-2008).

The data retrieved included biodata such as age, sex, occupation, aetio-pathological profile which includes presenting complaint, duration of complaints, associated history of allergy, number of episode(s), associated condition(s), nasal obstruction, epistaxis, nasal discharge, loss of smell, site, bilateral or unilateral, clinical diagnosis, histological diagnosis and outcome.

Patients with clinical diagnosis of benign lesion like inflammatory polyps had intranasal polypectomy with intranasal antrostomy while others had examination under anaesthesia (EUA) with incisional and excisional biopsy. Patients with clinical diagnosis of malignant nasal masses had examination under anaesthesia and tumour biopsy and some had nasal clearance in advanced disease to reduce tumour bulk and provide biopsy specimen.

All masses excised were subjected to histological examination. All the data were entered into the SPSS version 11.0 computer soft ware for analysis and results presented in tables and figures.

## Results

**Figure 1:**
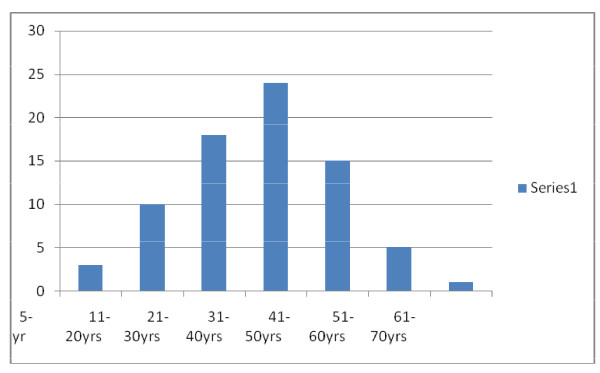
**Age - Frequency bar chart distribution**.

A total of 84 patients had SNM in the study period however only 76 patients had complete data for analysis which form the basis for this report. The age range from 5-64 yrs with a mean age of 33.3 yr median and modal age of 35.00 (SD = 13.1 ± 1.5). Majority of the patients were in the age groups 21-50 yrs (Figure 1). There were 34 males and 42 females with M: F ratio of 1:1.2.

About 31% of the patients that presented were either pupils or students, 30.1% were self employed, 21.5% were civil servants and 17.4% were housewives.

The main presenting symptoms are nasal blockage 97.4%, rhinorrhea 94.7%, allergic symptoms 52.6%, anosmia 34.6% others are as in Table 1. Duration before presentation was within 1 to 360 months with a mean duration of 40 months.

**Table 1:** Symptoms of presentation

Symptoms	Frequency (%)
Allergy	40 (52.6)
Epistaxis	23 (30.3)
Nasal blockage	74 (97.4)
Rhinorrhea	72 (94.7)
Anosmia	24 (34.6)
Asthma	6 (7.9)
Facial pain	7 (9.2)
Ophthalmic symptoms	12 (15.8)
Otological symptoms	26 (34.2)
Oropharyngeal symptoms	21 (27.6)
Recurrence of nasal masses	11 (14.5)

Sinonasal masses were found to be bilateral in 34(44.7%), left sided in 24(31.6%) and right sided in 18(23.7%) patients.

The clinical diagnosis in most of the cases correlates with the post operative histological diagnosis. The clinical diagnosis were simple nasal polyp in 47(61.8%) Out of the 47 with ethmoidal polyp 31 were male and 16 were females with M: F ratio of 2:1.

Antrochoanal polyp occurred in 10(13.2%) of the total sinonasal masses and common among those less than 20 yrs (60%), inverted papilloma 5(6.6% of the total sinonasal masses) with M: F ratio of 1:1.5, recurrent nasal polyp in pregnancy 4(5.3%) others as in table 2

**Table 2:** Clinical Diagnosis

Clinical diagnosis	Frequency (%)		
	Bilateral	Left	Right
Simple nasal polyp	30 (39.6)	11 (14.5)	6 (7.9)
Antrochoanal polyp	4 (5.3)	2 (2.6)	4 (5.3)
Inverted papilloma		1 (1.3)	4 (5.3)
Recurrent Nasal polyp in pregnancy		4 (5.3)	
Recurrent Simple nasal polyp		3 (4.0)	
Allergic nasal polyp	2 (2.6)		
Ethmoidal polyp	1 (1.3)		
Fungal sinusitis with polyp		1 (1.3)	
Nasal papilloma		1 (1.3)	
Nasal granuloma		1 (1.3)	
Simple Nasal polyp in bronchial asthma		1 (1.3)	

Histological diagnosis showed that 59 (77.6%) were benign, 2 (2.6%) were malignant. 15 (19.7%) patients did not have a histological diagnosis as they were either treated medically or lost to follow up.

**Figure 2:**
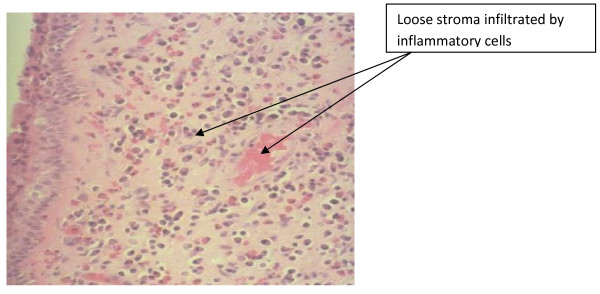
**Photomicrograph of nasal polyposis showing an edematous mass with loose stroma, infiltrated by inflammatory cells. H&E stain × 100**.

Histopathological diagnosis of the various sinonasal masses showed simple inflammatory nasal polyp in 28 (36.8%), inverted papilloma in 11 (14.5%), allergic nasal polyp 8 (13.1%), fibroepithelia polyp in 8 (10.6%), plasmacytoma 4 (6.6%) nasal capillary hemangioma 2 (2.6%), See Table 3. An Histopathological micrograph of the nasal polyposis showing an edematous mass with loose stroma, infiltrated by inflammatory cells. H&E stain × 100 as seen in figure 2.

**Table 3:** Histological diagnosis

Histological types	Frequency (%)
Simple inflammatory nasal polyp	35 (45.9%)
Simple allergic nasal polyp	10 (13.1%)
Inverted papilloma	14 (18%)
Capillary hemangioma	1 (1.3%)
Missing	21 (27.6%)

## Discussion

The nose and paranasal sinuses are involved in a wide variety of pathological conditions. Macroscopically simple nasal polyps are pale bags of non specific eosinophilic, oedematous, hyperplastic, sinonasal masses, they are most often bilateral, and indeed any unilateral lesion should be considered as a neoplasia, benign or malignant. The frequency of SNM increases with age similar to findings in our study, peaking in individuals aged 50 years or more [6], however our study showed a peak incidence of 33years which is relatively lower than findings by other previous workers [6].

There is a high incidence of benign non-neoplastic lesions in our study, constituting about 77.6% of cases while 2.6% were malignant and 19.7% had no pathologic diagnosis. Simple nasal polyps and antrochoanal polyps were the most common non-neoplastic sinonasal masses in this study forming up to 57(75%). Simple nasal polyps are uncommon in children under 10years of age in contrast to antrachoanal polyps and are similar to findings in our study, while another study reported it to be a presenting feature of cystic fibrosis [2]; however the least finding was among the elderly from our study.

Nasal polyp seems to occur more often in men, and their prevalence increases in both sexes with age from previous reports [6,7], our study showed higher preponderance among the females than males which is at variance with previous reports [2,5,7], this may probably be related to hormonal differences [8] but this was not classically established by our study. Antrochoanal polyps and inverted papilloma were found to be commoner in females than males which are similar to findings from previous reports [2,5,7]. Recurrence were observed in simple nasal polyps in 4 pregnant patients and in 3 other patients within 6 to 10 months of nasal polypectomy while some studies have recorded a recurrence rate as high as 29-53% [9].

A familial history has been reported in 14% of patients with NPs in an uncontrolled study [3] however this is at variance with our study with a higher value of 28.9% of patients with a family history of NPs. The hospital prevalence of sinonasal masses was estimated to be 1.2% as the total number of patients seen over this period is estimated to be 6333. This is still within the range found by other workers on this subject [4].

Occupation has been observed not to be a risk factor from our study as majority of the patients reviewed were students of different levels, then the self employed, civil servants and the least was unemployed full time housewives.

Earliest presentation was within one month with an average of 40months duration before presentation this may be due to patient's visit to non-specialists who have been offering palliative conservative treatment and are only referred after treatment failure.

The common symptoms and signs of sinonasal masses found in our study were nasal obstruction [2,10-12], rhinorrhea, feeling of nasal mass, epistaxis [7], loss of smell and voice changes [10] however majority of our patients presented with nasal obstruction and rhinorrhea which compares favourably with findings from other studies [2,6,7,10,11]. Epistaxis was noticed in 30.3% of the patients most of whom were 41years and above which should give a suspicion of neoplastic changes [2,6-8,11]. Symptoms of allergy such as rhinorrhea, itchy nostrils, excessive sneezing were noticed in more than 50% of the patients which support the fact that allergy still plays a major role in nasal polyp in our environment, however no demonstrable allergic confirmation were found from the records which is one of the deficiency of this study.

Only 18% of the records had documentation relating to voice changes which is one of the major complaints or observation in patients with sinonasal mass and this is usually more of a hyponasal speech which is in support of previous reports [2,6,10]. In addition 31.6% of the patients had anosmia based on symptoms which may be associated with taste changes which are characteristic of the symptoms [7] which was not volunteered from records due to the retrospective nature of our study. History of asthma was the least among our patients, this was at variance with a study by settipane et al [5] which reported that one third of patients with nasal polyp have asthma and only 7% of patients with asthma have nasal polyp which is comparable with our report of 7.9%.

Examination revealed bilateral sinonasal masses in 44.7% and unilateral in 55.3% out of which 31.6% were found on the right side and 23.6% on the left nasal cavity no reason could be deduced for this in our study. Nasal polyposis are invariably bilateral as noted in less than half of our patients similar to a previous report [13] and when unilateral as noted above requires histological examination to exclude malignancy or other pathology such as inverted papilloma [13] which was the commonest intermediate tumor recorded in our study. It was found to be commoner in females and this is similar to previous reports, even though it is a benign tumor the tendency towards malignant transformation is high and treatment is usually surgical excision and studies have found a recurrence rate as high as 50% after treatment. They are insensitive to palpation and rarely bleed [14,7,15].

About 5.3% of our patients had antrochoanal polyp. This was found more among the younger age group and its treatment is also via surgical excision and delivery via the nasopharynx. Pathological assessment of the nasal polyp showed that more than three-quarters of the nasal masses were benign in nature and this may be due to reduced risk of exposure to carcinogenic agents from wood work, boot and shoe work, furniture making and reduce exposure to environmental hydrocarbons in our series as documented in other reports [16] as majority of our patients were civil servants, students and those that are self employed trade in provisions, cloths and other materials non carcinogenic. About 2.6% of the patients had malignant nasal polyp and in almost one fifth of the population studied, some responded to medical treatment with steroid spray but were lost to follow up while some did not return their histological results after per-nasal biopsy was taken.

Out of the histological result available 45.9% were simple inflammatory polyp which is a benign lesion and most responded to surgical excision and follow up while 13.1% had allergic nasal polyp which showed evidence of high eosinophils in contrast to a previous study that reported allergic nasal polyp to be the commonest [6]. Eighteen percent had inverted papilloma, although it is a rare tumor occurring in approximately 0.5% of the nasal tumors thus representing about 4% of all nasal polyps but our study revealed a higher value of 18% [17], most of whom also had surgical excision (nasal polypectomy) and follow up. Out of the patients with inverted papilloma who were operated recurrence of polyp was noticed in 36% lower than that recorded by Buchwald et al [18], these patients were offered re- excision with referral for post operative chemo-radiotherapy. Other histological variants are as noted in Table 3 below.

On the treatment offered the patient a good number, 72.4% had surgical excision while 9.2% had medical treatment with nasal topical steroid spray with remission of the sinonasal masses which previous literature have found to be of value and safe in both allergic and non-specific rhinitis [9,19,20]. In this study, 1.3% of the patients had nasal steroid spray with minimal relieve of nasal obstruction but without remission of the sinonasal mass. These patients however declined surgery but were subsequently lost to follow up. A total of 17.1% patients were lost to follow up.

## Conclusion

Sinonasal masses are still thought to be a simple problem in our environment. The need for early recognition and referral to the ENT surgeon needs to be advocated among the primary care physicians as well as continuing medical education for the primary care physician on the care of sinonasal masses.

Nasal obstruction and rhinorrhea are the commonest symptom of presentation, bilateral is likely to be benign and commoner on the left side than the right side and simple inflammatory nasal polyp is still the commonest histological pattern seen in our environment.

For benign tumor surgery is still the best modality of treatment and in case of recurrence in unilateral nasal masses a suspicion of malignant transformation should be envisaged.

## Competing interests

The authors declare that they have no competing interests.

## Authors' contributions

AB: Conceived of this work, performed literature search, collected and analyzed data and reviewed the manuscript. OAA: Performed literature search, collected and analyzed data and prepared the manuscript. AAA: Collected and analyzed data and reviewed the manuscript critically for important intellectual content. AMK: Collected and analyzed data and reviewed the manuscript. BMA: Reviewed the manuscript and have given final approval of the version to be published.

All authors have read and approved the final manuscript for publication.

## References

[B1] Wright J History of laryngology and rhinology 1893 St Louis: Lea and Febiger 57 9

[B2] Newton J R Ah-See K W A review of nasal polyposis, Therapeutics and Clinical Risk Management 2008 4 2 507 512 10.2147/tcrm.s2379 PMC2504067 18728843

[B3] Hedman J Kaprio J Poussa T Prevalence of asthma, aspirin intolerance, nasal polyposis and chronic obstructive pulmonary disease in a population-based study Int J Epidemiol 1999 28 717 22 10.1093/ije/28.4.717 10480701

[B4] Laren PL Tos M Anatomic site of origin of nasal polyps: endoscopic nasal and paranasal sinus surgery as a screening method for nasal polyps in autopsy material Rhinology 1994 33 185 8 8919208

[B5] Settipane GA Epidemiology of nasal polyps Allergy Asthma Proc 1996 17 231 6 10.2500/108854196778662246 8922141

[B6] Lund VJ Diagnosis and treatment of nasal polyps BMJ 1995 311 1411 1414 8520278 10.1136/bmj.311.7017.1411 PMC2544406

[B7] Drake-Lee AB Kerr AG, Mackay AS, Bull TR Nasal polyps Scott-Brown's Otolaryngology 1997 4 7 Rhinology, Oxford: Butterworth-Heinneman 4/10/1-16.

[B8] Hillman E J Otolaryngologic manifestations of pregnancy - The Baylor College of Medicine in Houston, Texas Grand Rounds Archive 1995

[B9] Larsen K Tos M Clinical course of patients with primary nasal polyps Acta Otolaryngol (Stockh) 1994 114 556 9 10.3109/00016489409126104 7825441

[B10] Martin GF Lessening the Misery of Nasal Polyps Can Fam Physian 1991 37 1441 1444 PMC2145404 21229039

[B11] Mgbor N Onuigbo WLB Inverted papilloma of the nose and paranasal sinuses J Coll Med 2003 8 1 33 35

[B12] Fasunla AJ Lasisi AO Sinonasal malignancies: a 10-year review in a tertiary health institution J Natl Med Assoc 2007 99 12 1407 10 18229778 PMC2575924

[B13] Drake-Lee AB Nasal polyps Hospital Med 2004 65 264 7 10.12968/hosp.2004.65.5.13699 15176141

[B14] Fokkens W Lund V Mullol J European Position Paper on Rhinosinusitis and Nasal Polyps Group. European position paper on rhinosinusitis and nasal polyps. a summary for otorhinolaryngologists Rhinology 2007 45 97 101 EP3OS 17708455

[B15] Becker SS Surgical management of polyps in the treatment of nasal airway obstruction Otolaryngol Clin North Am 2009 42 2 377 85 10.1016/j.otc.2009.01.002 19328899

[B16] Cody DT II DeSanto LW Charles Cummings CW, Frederickson JM, Harker LA, Krause CJ, Richardson M, Schuller DE Neoplasms of the Nasal Cavity in Otolaryngology Head and Neck Surgery 1999 47 3 2 885

[B17] Mishra D Singh R Saxena R A Study On The Clinical Profile And Management Of Inverted Papilloma The Internet Journal of Otorhinolaryngology 2009 10 2

[B18] Buchwald C Fransman MB Tos M sinonasal papilloma Laryngoscope 1995 105 10 72 79 10.1288/00005537-199501000-00016 7837917

[B19] Pedersen CB Mygind N Sorensen H Prytz S Long-term treatment of nasal polyps with beclomethasone dipropionate aerosol Acta Otolarygol 1976 82 256 9 10.3109/00016487609120898 790891

[B20] Martin GF Pharmacology of nasal medication:an update Can Fam Physician 1988 34 2706 9 20469495 PMC2218146

